# Improved glycaemic control by switching from insulin NPH to insulin glargine: a retrospective observational study

**DOI:** 10.1186/1475-2840-8-3

**Published:** 2009-01-19

**Authors:** Peter Sharplin, Jason Gordon, John R Peters, Anthony P Tetlow, Andrea J Longman, Philip McEwan

**Affiliations:** 1 CHKS Health Economics Unit, Health Park, Cardiff, UK; 2 Department of Medicine, School of Medicine, Cardiff University, Cardiff, UK

## Abstract

**Background:**

Insulin glargine (glargine) and insulin NPH (NPH) are two basal insulin treatments. This study investigated the effect on glycaemic control of switching from a NPH-based regimen to a glargine-based regimen in 701 patients with type 1 (n= 304) or type 2 (n= 397) diabetes, using unselected primary care data.

**Methods:**

Data for this retrospective observational study were extracted from a UK primary care database (The Health Improvement Network). Patients were required to have at least 12 months of data before and after switching from NPH to glargine. The principal analysis was the change in HbA_1c _after 12 months treatment with glargine; secondary analyses included change in weight and total daily insulin dose. Inconsistent reporting of hypoglycemic episodes precludes reliable reporting of this outcome. Multivariate analyses were used to adjust for baseline characteristics and confounding variables.

**Results:**

After adjustment, both diabetic cohorts showed statistically significant reductions in mean HbA_1c _12 months after the switch, by 0.38% (p < 0.001) in type 1 patients and 0.31% (p < 0.001) in type 2 patients. Improvement in HbA_1c _was positively correlated with baseline HbA_1c_; patients with baseline HbA_1c _≥ 8% had reductions of 0.57% (p < 0.001) and 0.47% (p < 0.001), respectively. There was no significant change in weight or total daily insulin dose while on glargine. The majority of patients received a basal-bolus regimen prior to and after the switch (mean 79.3% before and 77.2% after switch in type 1 patients, and 80.4% and 76.8%, respectively in type 2 patients, p > 0.05).

**Conclusion:**

In routine clinical practice, switching from NPH to glargine provides the opportunity for improving glycaemic control in diabetes patients inadequately controlled by NPH.

## Background

Tight glycaemic control is a mandatory component of diabetes care given proven beneficial effects on the risk of vascular complications [1,2]. Current UK, European and US guidelines [3-9] recommend a target for glycated haemoglobin (HbA_1c_) between 6.5% and 7.5%. Insulin represents the cornerstone of care for achieving this target in patients with type 1 diabetes, and is also indicated in type 2 diabetes patients with suboptimal glycaemic control despite increasingly aggressive therapy with oral antidiabetic drugs (OADs) in addition to lifestyle changes.

In clinical practice, concerns about the initiation of insulin therapy contribute to less than optimal glycaemic control. As well, titration of insulin so as to achieve accepted targets for HbA_1c _while minimising the risk of hypoglycaemia and weight gain, may be problematic with conventional insulin regimens. The pharmacokinetic profile of short-acting basal insulins such as Neutral Protamine Hagedorn (NPH) are less than satisfactory for achieving a sustained duration of action. In contrast, more prolonged and predictable absorption is observed after administration of insulin glargine (glargine, Lantus^®^), the first long-acting basal analogue product. Following injection, glargine precipitates into the subcutaneous tissue and then dissociates slowly to form monomers that enter the circulation in a controlled fashion for 24 hours [10]. In clinical trials in patients with type 1 or type 2 diabetes, glargine was shown to be as effective as NPH insulin in lowering HbA_1c _levels [11,12] and was also associated with significantly fewer episodes of symptomatic and nocturnal hypoglycaemia [13]. In addition, most patients were adequately controlled by a once daily injection, due to the more prolonged and stable mechanism of action [14,15], leading to improved patient satisfaction [16]. Given the need to balance glycaemic control with the risk of hypoglycaemia, glargine may therefore offer benefits over NPH [17].

It is recognised that findings from randomised clinical trials are not necessarily representative of everyday clinical practice, particularly in respect of patient populations and adherence to medication [18,19]. Several observational studies [20-24] have attempted to address this issue. While these studies suggested that the use of glargine improved glycaemia control compared with NPH, deficiencies in patient selection and control for 'regression to the mean' effects and potential confounding variables make these analyses difficult to interpret. Therefore, we conducted a retrospective observational study based on a large patient cohort derived from primary care practices in the UK to assess the impact on HbA_1c_, weight and insulin use of switching from NPH to glargine.

## Methods

### Data source

The data were sourced from a large national computerised medical record database known as The Health Improvement Network (THIN), which includes data from 211 UK primary care practices collected over a 15 year period from about 5 million patients, of whom 2.34 million were actively registered with a practice and prospectively followed [25]. The THIN database is not supported by any industrial sponsor, nor biased towards any particular disease group. THIN data on patient demographics, medical history, test results and drug treatments are collected in a non-interventional manner during daily record keeping within the primary care practice. To ensure confidentiality of patient information, the data are anonymised at the collection stage using encrypted identifiers for the physician and individual. Ethical approval for this study was obtained from the London Multiple Research Ethics Committee (Number 06/MRE02/32) before commencing data extraction.

From data collected between July 2002 and December 2005, as described previously [26,27], 137,258 patients were identified as diabetic based on a relevant medical diagnosis via the Read code system [28], or prescription of OAD. Diagnosis of diabetes was attributed in a stepwise manner. For patients without a specific diagnosis of diabetes, a diagnosis of type 2 diabetes was attributed if the subject had received any non-insulin, diabetes-related medication. Overall, 90% of patients were identified with type 2 diabetes.

Individuals were included in the current analysis if they had 1) been prescribed NPH for at least 12 months prior to switching to a glargine-based regimen, and 2) continued treatment with glargine for at least 12 months. Treatment with OADs and/or bolus of prandial insulin was permitted in addition to basal glargine. For this study we also extracted information on associated comorbidities including: myocardial infarction, stroke, peripheral vascular disease, peripheral neuropathy, nephropathy and retinopathy. Use of analogue and human prandial insulins could not be distinguished from information collected.

### Study design and outcome measures

This was a retrospective, 24-month non-randomised, observational study. The principal analysis was glycaemic control as assessed by HbA_1c_. Measurements were performed locally in each centre and mean HbA_1c _values were calculated every 3 months before and after switching insulin therapy using actual or linearly interpolated values. Although much of the UK is currently HbA_1c _DCCT-aligned, and primary care practices use National Health Service hospital laboratories which are members of quality assurance schemes, the degree of standardisation at the time of data collection (2002–2006) is not known. However, our study depends on change in HbA_1c _and will thus be less sensitive to differences in calibration between assays. Secondary analyses included mean change in weight (kg) calculated as for HbA_1c_, mean change in prescribed daily insulin dose calculated as units prescribed divided by the number of days covered by the prescription, the proportion of patients using bolus prandial insulin, and the percentage of patients achieving defined HbA_1c _levels. Self-reported episodes of hypoglycaemia were recorded by general practitioners during each 3 monthly interval.

### Statistical methods

Linear interpolation of missing data was performed where a patient had at least 2 data measurement during each 12 month period (prior to and following switch) and data was not missing during two consecutive 3 monthly intervals. Unadjusted results for the principle (HbA_1c_) and secondary analyses used linearly interpolated data and were summarised using descriptive statistics. For the unadjusted results the mean change during the 12 month prior to and following the switch was calculated. Comparisons were performed using paired t-tests. Graphical analyses were based on linearly interpolated data, which provides a clearer graphical interpretation of the results.

For the principle analysis of change in HbA_1c _a multivariate analysis using actual patient data was performed. Actual patient data was preferred over interpolated values; multivariate models constructed using the later showing no appreciable effect on the model specification or statistical inference. Data was assessed in a linear mixed effect modelling framework, adjusting for repeated measures per patient over time, with change in HbA_1c _relative to time of insulin initiation as the dependent variable with the following pre-defined (fixed-effects) exploratory covariates; age, weight, sex, type of diabetes, number of OADs used prior to commencing insulin, number of OADs used in combination with insulin at initiation, disease duration, presence of hypoglycaemia and associated co-morbidities during the study.

Multivariate models were developed with SPSS for Windows (version 8; SPSS, Chicago, IL, USA) using a backward stepwise approach; non-significant variables at the 5% level were excluded. Sensitivity analyses were performed to consider treatment effect by baseline HbA_1c _levels. Secondary endpoints were summarised descriptively.

## Results

### Subjects and baseline characteristics

A total of 701 patients, 304 (43%) with type 1 diabetes and 397 (57%) with type 2 diabetes, were included in the study. Mean HbA_1c _at baseline before switching was similar in each group (8.8% and 8.9%, respectively) (Table 1). In total, 22% of patients with type 2 diabetes received an OAD prior to the switch. The majority of patients received a basal-bolus NPH-based regimen prior to the switch (Table 1).

**Table 1:** Baseline characteristics of patients switching from NPH to glargine

	**Type 1**	**Type 2**
n (%)	304 (43.4)	397 (56.6)
% male	47.4	52.4
Age (years)*	34.8 ± 13.2	48.2 ± 13.8
Weight (kg)	76.6 ± 14.7	82.9 ± 16.7
Baseline HbA_1c_*†	8.8 ± 1.5	8.9 ± 1.4
Duration of diabetes (years) ‡	13.4 ± 10.0	10.0 ± 8.6
Number of co-morbidities*§	1.7 ± 1.3	1.9 ± 1.3
Proportion of patients receiving OAD(s) (%)	2	22
OAD per patient before starting insulin*¶	0.0 ± 0.0	0.2 ± 0.4
Proportion of patients receiving bolus doses (%)		
At any point in prior 12 months	97	91
In prior 3 months	79	80
Hypoglycaemia episodes in a 3 month period*||		
No. episodes	36	40
Mean no. episodes per patient	0.12	0.10

OAD = oral antidiabetic drugs; HbA_1c _= glycated haemoglobin. Data are mean ± SD unless indicated.

*Significant variables investigated in multivariate analysis.

†Data missing for 30 patients with type 1 diabetes and 39 patients with type 2 diabetes.

‡Data missing for 3 patients with type 1 diabetes and 8 patients with type 2 diabetes.

§Includes myocardial infarction, stroke, peripheral vascular disease, neuropathy, nephropathy, and retinopathy. Data available for 57 patients with type 1 diabetes and 76 patients with type 2 diabetes.

¶Number of oral diabetic treatments (e.g. metformin, sulfonylureas) prescribed prior to commencing insulin. Data missing for 100 patients with type 2 diabetes.

||Number of hypoglycaemic episodes reported during the 3 month period prior to switch. Hypoglycaemic episodes were reported in 30 patients with type 1 diabetes and 33 patients with type 2 diabetes.

### Change in HbA_1c_

**Figure 1:**
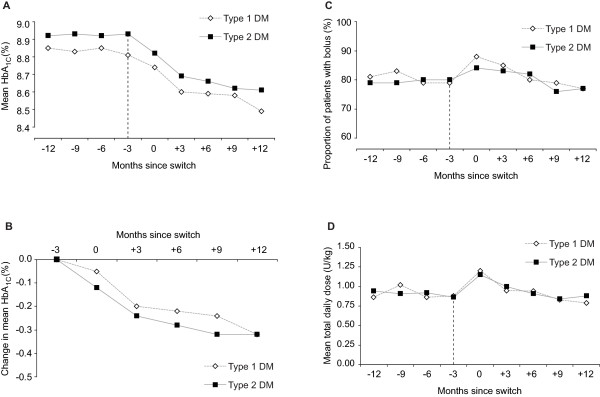
**Mean HbA_1c _12 months before and after switching from NPH to glargine (A), mean change in HbA_1c _after switch (B), use of bolus insulin before and after switch (C) and total daily insulin dose before and after switch (D) (unadjusted data)**. The last measurement for NPH is at -3 months (indicated by vertical dotted line). During period -12 m to -3 m patients are taking NPH only. During 3 month switch time point (0 months) patients may be prescribed NPH or glargine. During period +3 m to +12 m patients are only prescribed glargine. Linearly interpolated data were used to graphically depict the change in each parameter. Linearly interpolated data affords a clearer graphical interpretation but may bias estimates of variance; as such error bars (95% confidence intervals for the means) are not reported. Total daily insulin dose was calculated according to the number of units prescribed divided by the number of days covered by the prescription.

Mean HbA_1c _was stable and similar in both type 1 and type 2 diabetic cohorts in the 12 months prior to the switch to glargine (Figure 1A). After 12 months on glargine, unadjusted mean HbA_1c _levels decreased significantly by 0.32% in both cohorts (from 8.81% at baseline to 8.49%, p = 0.003 in the type 1 cohort and from 8.93% to 8.61%, p = 0.0004 in the type 2 cohort) (Figures 1B and 1C). After adjustment for significant demographic and clinical covariates, including age, weight, baseline HbA_1c_, concomitant bolus usage and hypoglycaemia, the decrease in mean HbA_1c _over 12 months was 0.38% (p < 0.001) in type 1 diabetes patients and 0.31% (p < 0.001) in type 2 diabetes patients. Sensitivity analyses showed that improvement in HbA_1c _after switching was positively correlated with baseline HbA_1c_. Patients with baseline HbA_1c _≥ 10% had the greatest reduction in mean HbA_1c _(-1.07%, p < 0.001 in the type 1 diabetes cohort and -0.97%, p < 0.001 in the type 2 diabetes cohort) (Table 2). Age did not significantly influence the change in HbA_1c _following the switch (data not shown).

**Table 2:** Adjusted HbA_1c _reduction over 12 months in patients switching from NPH to glargine*

	**Type 1 (n = 304)**			**Type 2 (n = 397)**		
		
**Variable**	**No.**	**Δ HbA_1c _(%)**	**p-value**†	**No.**	**Δ HbA_1c _(%)**	**p-value**†
Overall	304	-0.38	<0.001	397	-0.31	<0.001
HbA_1c _by baseline level						
*≥ 7%*	259	-0.42	<0.001	327	-0.36	<0.001
*≥ 8%*	186	-0.57	<0.001	271	-0.47	<0.001
*≥ 9%*	107	-0.96	<0.001	162	-0.69	<0.001
*≥ 10%*	53	-1.07	<0.001	71	-0.97	<0.001

*Adjusted for significantly correlated demographic and clinical covariates including concomitant use of bolus, age, weight, hypoglycaemia, and baseline HbA_1c _(in the 3 months prior to insulin initiation).

†p-values by the paired t-test for difference in mean HbA_1c _following switch from NPH to glargine.

### Proportion of patients reaching HbA_1c _levels

In both cohorts, 31% of patients achieved an HbA_1c _level of 7% within 12 months of switching from NPH to glargine. Overall, 18% of patients with type 1 diabetes and 20% with type 2 diabetes achieved a reduction in HbA_1c _of ≥ 1%.

### Episodes of hypoglycaemia

During the 12 months prior to the switch from NPH to glargine, 115 hypoglycaemic episodes were reported by the patients with type 1 diabetes (0.38 episodes per patient/year), while 131 episodes were reported by patients with type 2 diabetes (0.33 episodes per patient/year). After switching to glargine, the respective data were 244 episodes for the patients with type 1 diabetes (0.80 episodes per patient/year) and 286 episodes for the patients with type 2 diabetes (0.72 episodes per patient/year) during the following 12 months.

### Change in use of bolus, total insulin usage and weight

There were no significant changes over the 12 months following the switch with respect to the use of prandial insulin boluses (mean 79.3 ± 4.3% before and 77.2 ± 4.9% after switching in type 1 patients, and 80.4 ± 4.0% and 76.8 ± 4.3%, respectively in type 2 patients, p > 0.05, Figure 1C) or the total daily (glargine + prandial) insulin dose (1.0 ± 2.0 U/kg before and 0.89 ± 1.48 U/kg after switching in type 1 patients, and 0.96 ± 1.82 U/kg and 0.97 ± 1.44 U/kg in type 2 patients, p > 0.05, Figure 1D). While there was an initial increase in the calculated total daily dose of glargine (about 20 U per day) in the first 6 months after the switch in each cohort, this subsequently stabilised over the next 6 months. This small initial increase may be due a "stock building effect", whereby patients are initially prescribed additional insulin doses to allow them to store a "security stock" when starting the treatment with their new insulin (daily dose of insulin was calculated from the quantity of insulin prescribed at each visit). The use of OADs remained unchanged after the switch, with quarterly estimates indicating that between 20% and 22% of patients received OADs. Switching from NPH to glargine was not associated with any significant change in weight (mean weight 76.7 ± 1.8 kg before and 77.8 ± 2.2 kg 12 months after switching in type 1 patients, and 82.9 ± 1.7 kg and 82.7 ± 2.0 kg, respectively in type 2 patients, p > 0.05).

## Discussion

The results of this retrospective observational study show that patients with type 1 or type 2 diabetes, who switch from a predominantly basal-bolus NPH-based regimen to a glargine-based basal-bolus regimen derive significant improvement in glycaemic control (adjusted mean decrease in HbA_1c _by 0.38% and 0.31%, respectively) within 12 months, without any significant increase in weight, use of prandial boluses, total insulin dose or OAD intensity. These findings add to a weight of evidence supportive of improved glycaemic control when patients switch from other insulins, either NPH [29,30] or premixed insulins [31], to glargine. The relevance of our findings are strengthened by the nature and size of our sample (701 patients in an everyday clinical practice setting), the quality of baseline data (available for at least 12 months prior to switching from NPH to glargine), the completeness of follow-up data after switching (at least 3 monthly follow-up over 12 months), and the use of linear interpolation and multiple regression techniques in our analysis. Sensitivity analyses showed that the magnitude of improvement in HbA_1c _was greatest in patients with the poorest glycaemic control (mean adjusted decreases of 0.57 and 1.07% in type 1, and 0.47 and 0.97% in type 2 diabetes patients with baseline HbA_1c _levels ≥ 8% and ≥ 10%, respectively) (Table 2), and comparable with that reported in trials of patients newly commencing insulin therapy [32]. A reduction of 1% in HbA_1c _is associated with a 14% reduction in myocardial infarction, a 14% reduction in all-cause mortality, a 37% reduction in microvascular complications, and a 21% reduction in overall diabetic complications [33]. Thus, the additional reduction in HbA_1c _achieved by switching to a glargine-based regimen can be considered clinically meaningful, both in the overall population and the subgroups which had a ≥ 1% greater reduction, given the potential to translate to clinical outcomes benefits in the longer-term.

While we have highlighted the strengths of our study, we also acknowledge a number of potential limitations. First, we recognise that retrospective observational studies do not provide the same robust level of evidence as randomised controlled trials. In our study, the decision to switch from NPH to glargine treatment was not standardised but instead based on the judgement of the individual treating clinician. The data were collected from a large number of primary practice units which is likely to introduce a considerable level of heterogeneity to the main findings. As well, the level of data collection did not permit investigation of potential influences on glycaemic control including differences in racial background [23], body mass index [29,34], frequency of NPH and glargine use (once vs. twice daily) [35], concurrent use of OADs with glargine [36], time of administration of glargine (morning vs. bedtime) [37,38] and patient compliance [39,40]. Second, it was not possible to reliably assess data concerning hypoglycaemic episodes in patients who switch from a NPH-based regimen to a glargine-based regimen. A lack of consistency in recording these data in the THIN database meant that there is strong likelihood that we underestimated the real incidence of hypoglycaemia and only captured the most severe episodes. The incidence of hypoglycaemia may also reflect the self-reporting methods used, as patients and physicians were not requested to provide specific details of each episode. The limitations of the database also did not allow assessment of the nature (e.g. nocturnal) or severity of hypoglycaemic episodes before and after the switch. A higher number of hypoglycaemic episodes were noted after the switch from NPH to glargine. However, the overall rate of hypoglycaemia was low so the results should interpreted with caution. Should this trend be real, the significant improvement in glycaemic control observed with glargine in both diabetic cohorts may have made patients more susceptible to episodes of hypoglycaemia.

Despite these shortcomings, observational studies such as the current report are generally regarded as an ideal approach to assess the actual health outcomes of patients in routine care, more so than randomised controlled trials. This is because the level of care patients receive in clinical trials is often of a higher standard and not representative of that provided in daily clinical practice [18,19]. As well, clinical trials usually have limited scope for titration of other glucose-lowering drugs, a relatively short observational period, and potential for population bias which may prevent extrapolation of their findings to everyday practice [41,42]. In the current study, there were no limitations regarding patient inclusion criteria or clinical criteria for switching insulin treatment, which is likely to widen the implications of our findings. Furthermore, while high rates of missing data and patient drop-outs are common criticisms of observational studies, the current study provided interpolated data for 90% of patients during the months preceding and immediately following the switch and for 67% 12 months later. Interpolated HbA_1c _data was used in 60% of patients. However, the main analysis performed in our study was based on the adjusted change in HbA_1c _which only used actual and complete data values. It is reassuring to note that our findings are supported by other analyses from different populations and geographic locations and using different methods of data collection [29].

The implications of these results for decision makers are that improvement in control of glycaemia, as measured by HbA_1c_, can be directly related to treatment efficacy and the costs of subsequent care. Using a discrete event simulation model, we recently reported that that for UK patients with sub-optimally controlled type 2 diabetes, taking into account effects on HbA_1c _and reduction in hypoglycaemia, glargine can be considered as a cost-effective treatment option with a cost per quality-adjusted life year of less than £10,000 [43]. In addition, preliminary data from a similar analysis using the THIN database indicates that for patients with type 1 or type 2 diabetes who switch from NPH to glargine, treatment with glargine is a cost-effective strategy according to conventional thresholds [44].

A noteworthy finding from our study is that despite dissemination of clinical guidelines for diabetes management, glycaemic control is still less than optimal in a large proportion of patients in primary practice in the UK. Approximately two-thirds of patients in the study failed to achieve an HbA_1c _level ≤ 7%. These data highlight the need for additional treatment strategies. These may include higher doses and more aggressive titration (in the current study, evidence that glargine had no significant effect on weight is suggestive of suboptimal treatment), greater use of OAD therapy in patients with type 2 diabetes (only 20% were prescribed OAD therapy at the time of the switch), as well as the use of educational programmes [45-48].

## Conclusion

This observational study suggests that in diabetes patients treated with NPH and with evidence of suboptimal efficacy and/or poor tolerability, switching to insulin glargine offers the opportunity for improved glycaemic control.

## Abbreviations

HbA_1c_: Glycated haemoglobin; OADs: oral antidiabetic drugs; NPH: Neutral Protamine Hagedorn; THIN: The Health Improvement Network.

## Competing interests

All authors were consultants to Sanofi-Aventis, had full access to all the data in the study and took responsibility for the decision to submit for publication.

## Authors' contributions

PS and JG performed the statistical analyses and drafted the manuscript. JP, APT, AL, and PM were involved in study design, coordination and data acquisition. All authors have read and approved the final manuscript.
